# From Obscurity to Prominence: IPMK’s Expanding Role in Cellular Signaling, Physiology, and Disease

**DOI:** 10.3390/biom15091266

**Published:** 2025-09-01

**Authors:** Subrata H. Mishra, Sujan Chatterjee, Loretta Viera-Preval, Prasun Guha

**Affiliations:** 1 Institute for Bioscience and Biotechnology Research, National Institute of Standards and Technology, University of Maryland, Rockville, MD 20850, USA; shmishra@umd.edu; 2 Nevada Institute of Personalized Medicine, University of Nevada, Las Vegas, NV 89154, USA; sujan.chatterjee@unlv.edu (S.C.); vierapre@unlv.nevada.edu (L.V.-P.); 3 School of Life Sciences, University of Nevada, Las Vegas, NV 89154, USA

**Keywords:** IPMK, inositol, PIP3, cell migration, physiology, DNA repair, mTOR, InsP, HOIP

## Abstract

Once a protein of relative obscurity, inositol polyphosphate multikinase (IPMK) emerged as a versatile and indispensable enzyme in cellular biology. With dual inositol and lipid kinase activities, IPMK generates pivotal signaling molecules such as InsP4 (inositol tetraphosphate), InsP5 (inositol pentaphosphate), and PIP3 (phosphoinositide 3,4,5-trisphosphate), positioning it as a critical regulator of cellular mechanisms. Initially identified in yeast and later recognized as essential for mammalian embryonic development, IPMK has transitioned from a niche interest to a focal point in studies of nutrient sensing, growth factor signaling, mRNA transport, and transcription regulation. Over two decades, multidisciplinary research has unveiled its far-reaching biological roles and implications in diverse diseases, including neurodegeneration, cancer, and inflammation. This review charts IPMK’s journey from obscurity to prominence, examining its structure–function relationships, cellular roles, and emerging physiological impacts, while highlighting its potential as a therapeutic target in human health and disease.

## 1. Introduction

**Figure 1:**
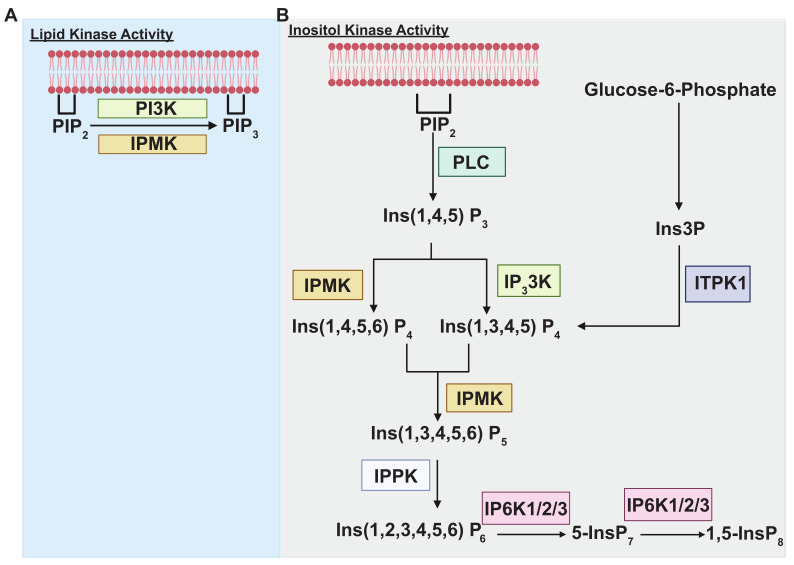
IPMK’s biochemical and enzymatic function. (**A**) IPMK exhibits lipid kinase activity, facilitating the conversion of PIP_2_ to PIP_3_ [15], which can also be catalyzed by class I PI3Kinase (PI3K). (**B**) In the HOIP pathway, Ins(1,4,5)P_3_ is generated from PIP_2_ via PLC [16]. This InsP_3_ can be further converted into Ins(1,4,5,6)P_4_ by IPMK or into Ins(1,3,4,5)P_4_ by IP_3_3K [16]. Additionally, glucose-6-phosphate can be transformed into Ins3P, which is subsequently utilized by ITPK1 to produce Ins(1,3,4,5)P_4_ [16]. Both Ins(1,4,5,6)P_4_ and Ins(1,3,4,5)P_4_ are then processed by IPMK to form Ins(1,3,4,5,6)P_5_ [16]. Finally, IPPK converts Ins(1,3,4,5,6)P_5_ into Ins(1,2,3,4,5,6)P_6_, which serves as a substrate for IP6K to generate 5-InsP7. The 5-InsP7 can subsequently be converted into 1,5-InsP8 [16].

The discovery of inositol 1,4,5-trisphosphate (InsP3) as a critical secondary messenger that stimulates intracellular calcium release [1] revolutionized our understanding of cellular signaling. Since Michael Berridge and colleagues’ seminal discovery in 1983 [2] that InsP3 mobilizes intracellular calcium release from the endoplasmic reticulum [1,2,3], extensive research has illuminated InsP3’s crucial roles in regulating diverse physiological processes. These include muscle contraction [4], synaptic transmission [5], and embryonic development [6]. Moreover, aberrant InsP3 signaling has been implicated in various pathological conditions, including cancer [7,8] and neurological disorders [9]. While the importance of InsP3 is well-established, a significant knowledge gap remains in our understanding of higher-order inositol phosphates (HOIPs) and their biological functions. The HOIP pathway, which uses InsP3 as a precursor, involves a series of phosphorylation events catalyzed by various kinases [10,11] (Figure 1). A key enzyme in this pathway is inositol polyphosphate multikinase (IPMK), a dual-specificity 6-/3-kinase that generates InsP4 and InsP5 species [11,12]. IPMK acts as a rate-limiting enzyme, with its loss of function significantly impacting the production of downstream HOIPs such as InsP4, InsP5, InsP6, InsP7, and InsP8 [13,14].

In recent years, IPMK and other enzymes involved in HOIP generation have garnered significant attention from researchers. IPMK, in particular, has emerged as a versatile regulator of cellular functions, exhibiting both kinase-dependent and independent roles. Its diverse functions span a wide range of cellular processes, including mRNA export [17,18], chromatin remodeling [19,20], transcription [21,22], nutrient sensing [23], and growth factor signaling [15]. The physiological significance of IPMK is highlighted by studies across various model organisms, where its deletion results in severe developmental defects [24,25]. Notably, among all enzymes in the HOIP pathway, IPMK stands out as the only one whose deletion leads to embryonic lethality in mice, underscoring its critical role in mammalian development [25]. This unique position of IPMK in the HOIP pathway emphasizes its importance in cellular homeostasis and developmental processes, making it a focal point for understanding the broader implications of inositol phosphate signaling in biology.

IPMK is a rare example of a protein with dual kinase properties. It functions as both an inositol kinase and a lipid kinase, capable of converting PIP2 (Phosphoinositide 4,5 bi phosphate) to PIP3 (Phosphoinositide 3,4,5 triphosphate) [26,27]. This dual functionality significantly expands IPMK’s regulatory role in cellular processes. Notably, research suggests that IPMK’s lipid kinase enzymatic (PI3-kinase) activity is essential for activating AKT [15], also known as PKB (protein kinase B), a crucial kinase involved in cell survival and proliferation.

Over the past two decades, research utilizing animal models and human mutation analyses has revealed IPMK’s involvement in a wide array of human diseases, including inflammatory disorders [28,29], obesity [30,31], Alzheimer’s disease [32], inflammatory bowel disease [33,34], and intestinal neuroendocrine tumors [35]. These findings highlight a complex regulatory mechanism 3 of IPMK in maintaining cellular homeostasis and its potential as a therapeutic target.

This review aims to provide a comprehensive overview of IPMK research, its structure–function relationships, and the complex mechanisms by which IPMK modulates mammalian signaling pathways and contributes to the pathogenesis of diverse human diseases.

## 2. IPMK Exhibits Both Inositol Kinase and Lipid Kinase Enzymatic Activity

### 2.1. Lipid Kinase Enzymatic Activity

IPMK uniquely serves as a lipid kinase (Figure 1A), utilizing Phosphatidylinositol 4,5-bisphosphate [PI (4,5) P2] (PIP2) as a substrate to generate [PI (3,4,5) P3] (PIP3). Both yeast and mammalian IPMK exhibit lipid kinase properties [15,26]. Interestingly, in contrast to class I phosphoinositide 3-kinase (PI3K), traditionally considered the primary cellular lipid kinase, generating multiple products such as PI (3,4,5) P3, PI (3,4) P2, and PI (3) P from lipid extracts [26,36], IPMK displays remarkable selectivity. Specifically, IPMK generates only PI (3,4,5) P3 as its product [26], highlighting its unique enzymatic specificity in lipid kinase activity.

### 2.2. Inositol Kinase Enzymatic Activity

In the HOIP pathway (Figure 1B), IPMK, along with Inositol-trisphosphate 3-kinase 1 (ITPK1), plays a crucial role in generating inositol polyphosphates (InsPs) from Inositol 1,4,5-trisphosphate (InsP3) [10,16]. Traditionally, InsP3, derived from Phosphatidylinositol 4,5-bisphosphate (PIP2) by phospholipase C (PLC), has been considered the primary precursor for InsP4 (InsP 1,4,5,6) production [37]. However, recent findings [38] suggest that ITPK1 can utilize Glucose-6-phosphate-derived InsP3 to generate InsP4 (InsP 1,3,4,5), and both isoforms of InsP4 are converted to InsP5 (InsP 1,3,4,5,6) exclusively by IPMK. Hence, IPMK can use either InsP3 or InsP4 to generate InsP5 (Ins 1,3,4,5,6). Subsequently, inositol pentakisphosphate 2-kinase (IPPK) converts InsP5 into InsP6 (Ins 1,2,3,4,5,6) [10]. InsP6 can undergo further phosphorylation to form InsP7/8 by InsP6 kinases 1/2/3 [10]. In mammalian cells, InsP5 and InsP6 are produced in significantly higher concentrations compared to other inositol phosphates [39,40], representing the highest products in the HOIP pathway.

Studies across various organisms, from yeast to mammalian cells, have demonstrated a significant decrease in both InsP5 and InsP6 production in *IPMK*-null or kinase-dead cells [12,14]. This reduction is primarily attributed to the decrease in InsP5 levels, which serves as the major precursor of InsP6 upon *IPMK* deletion, underscoring the critical role of IPMK in regulating HOIPs levels.

## 3. IPMK Structure and Function

### Protein Architecture

**Figure 2:**
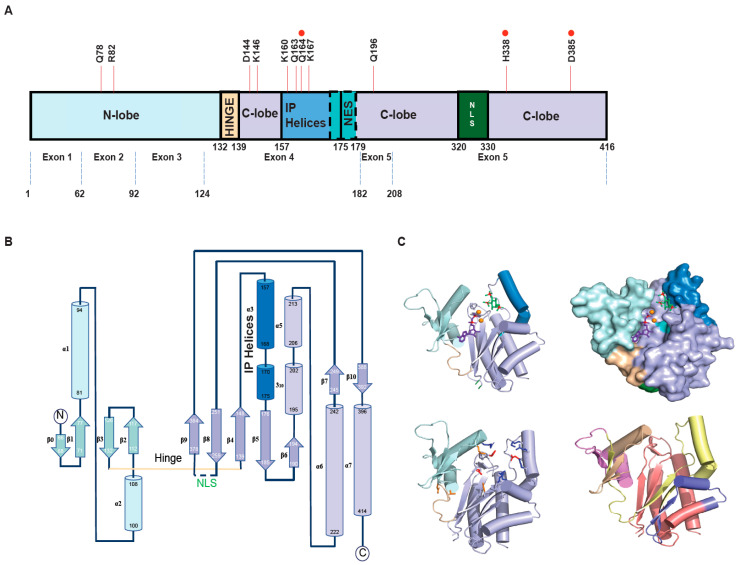
(**A**) Human IPMK (HsIPMK) protein’s domain and exon boundaries, and critical residues involved in catalysis. The IPMK sequence is primarily divided into an N-lobe and a C-lobe joined by a hinge region. The elements—IP helices, nuclear export signal (NES), and nuclear localization signal (NLS)—are all part of the C-lobe. The NES (nuclear export sequence) is marked by dotted lines as it overlaps (170–179) with the IP helices. Residues that are critically involved in IPMK catalytic activities are indicated by red lines. The red circles above certain residues indicate near-complete abrogation of catalytic activity when mutated. Color schemes are maintained for topology (**B**) and 3D structural views (**C**). (**B**) Topology map of HsIPMK (pdb id: 5w2g). The numbering for alpha helices or beta sheets is as published for the pdb id 5w2g [42]. (**C**) Three-dimensional structure of HsIPMK (pdb id: 5w2h) with bound substrates—ADP (purple), IP3 (green)—as a 3D cylinder and sheets model (top left) and as a surface model (top right). Mg^2+^ ions (orange spheres) and phosphates on ADP and IP3 are colored red. All IPMK color schemes are as in (**A**). The NLS (dark green) was replaced with a flexible linker to be able to crystallize this protein. Critical residues (Table 2) are displayed (bottom left) with residues proximal to ATP in orange and those to InsP3 in blue. The 3 residues in red, when mutated individually, result in a nearly inactive enzyme. The color scheme here () for the protein simply depicts N- and C-lobes and the hinge for simplicity. Lastly, exon boundaries (bottom right) are mapped onto the IPMK 3D protein structural model—exon 2 (beige), exon 3 (pink), exon 4 (lemon yellow), exon 5 (blue), and exon 6 (salmon red).

The human IPMK protein is a 416 amino acid-long protein comprising two pseudo-domains—an N-lobe and a C-lobe—joined by a hinge (Figure 2A). The N-lobe and the C-lobe interface form a groove-like cavity that harbors the catalytic site, which facilitates the transfer of phosphates from ATP to inositol head groups of PIP2 and InsP3, InsP4. The N-lobe is the smaller of the two lobes, with two α-helices and four anti-parallel β-strands forming a single β-sheet. The first β-strand (β0 in Figure 2B) is not always visible in all crystal structures owing to the disordered nature of the first roughly 60 amino acids [41,42], which has implications for interaction with other proteins (Table 1) as well as catalytic activity. The C-lobe, which constitutes the bulk of the IPMK residues, has six helices (five α-helices and one 3_10_ helices) and seven β-strands, which assemble into two β-sheets (Figure 2B, β4–6, 8, 9 and β7, 10). Inositol kinases achieve substrate specificity via a structural element called the IP helices, which in HsIPMK consists of an α-helix and the 3_10_ helix (α3 and α4 in Figure 2B). There are other structural features that have been discussed in the literature to compare IP kinases—G-loop, gatekeeper, R- and C-splines—but the elements discussed above provide a basic yet complete overview of the structural elements that guide catalysis. The N-lobe ATP grasp fold, along with the hinge, guides the ATP to transfer its γ phosphate to the InsP3 (1, 4, 5) cradled in the cavity created by the IP helices to yield InsP4 (1,3,4,5; 3-kinase activity), which is further phosphorylated to InsP5 (1,3,4,5, 6; 6-kinase activity). Currently, different groups have deposited crystal structures of human IPMK in the PDB database in its free form (pdb id: 5w2g, 6e7f) [41,42] bound to substrates (ADP, InsP3) [42] or inhibitors [43]. The protein constructs in these crystal structures deviate from the wild-type human IPMK sequence as they exclude the region encompassing the NLS region (residues 263–377 in pdb id 5w2g, 279–373 in pdb id 6e7f), which is replaced by a short artificial linker, to aid the crystallization of this protein. Both reported structures are in close agreement with a backbone root mean square deviation of 0.48 Å.

**Table 1:** IPMK interaction partners.

Protein	IPMK Interaction Regions	Verification Assay	Comments
**AMPK**	125–182 and 209–416	Dominant-negative	Exons 4 and 6; Phosphorylation of IPMK at Y174 facilitates IPMK-AMPK binding [44]
**CK2**	284–343	In vitro phosphorylation	CK2 phosphorylates IPMK at S284. An IPMK putative binding site (283–287) for Ck2 is predicted [45].
**CREB-binding protein (CBP)**	1–75	Dominant-negative	Mouse IPMK [22]
**Chromatin remodeling complex SWI/SNF**	93–124, 125–182, and 208–416	In vitro binding	The full-length IPMK was verified to bind the core subunits of SWI/SNF complex- SMARCB1/BRG1/BAF155 proteins, and IPMK exons 3, 4, and 6 interact with SMARCB1 Rpt1 and Rpt2 domains [20]
**Disheveled-3 (DVL3)**	1–77	In vitro binding	The binding of IPMK involves the PDZ (247–334) and the C-terminal tail (496–716) of Dvl3 [46]
**mTOR**	1–60	Dominant-negative	Exon 1 [23]
**raptor**	61–416	Dominant-negative	Interaction seen using 1–182 and 182–416 IPMK fragments but not 1–60 [23]
**Nrf2**	336–416	Pulldown analysis	Interactions were evaluated endogenously in liver and brain (cortex) lysates [47].
**p53**	125–184	Dominant-negative	Exon 4 [48]
**nuclear receptor** **SF-1 (NR5A1)/PIP2**	Full length	In vitro binding	IPMK binds SF-1/PIP2 only when PIP2 is incorporated into the hydrophobic ligand binding domain of SF-1 [21]
**Ulk1**	182–252	Dominant-negative	IPMK may act as a scaffold to link AMPK with ULK1 [14]
**SRF**	93–124, 125–182, and 208–416	Dominant-negative	Exons 3, 4, and 6 are involved in binding with the SRF DNA binding domain (MADS); exons 3 and 4 are verified as the dominant-negative structure [46]
**TRAF6**	93–124 and 208–416	Dominant-negative	Exons 4 &6. IPMK binds the N-terminal RING domain of TRAF6 (1–132) [29]
**TFEB**	262–377	In vitro binding	Positively charged residues in the IPMK NLS-320, 322, 323, 327, and 328 were mutated to verify TFEB interactions [49]

Note: Unless mentioned under Comments, IPMK interaction regions are for the human IPMK sequence.

The substrate binding pocket for HsIPMK is more constrained than its plant and yeast orthologs. HsIPMK has a unique proline loop harboring residues (Table 2) that coordinate with ATP, as well as the inositol substrate, and is absent in orthologs or IP3Ks and IP6Ks. Interestingly, the inositol substrate selection cavity of IPMK has more glutamines compared to arginine or lysines in other IP kinases. These glutamines and other residues critical to IPMK’s catalytic activity are noted in Table 2. The catalytic transfer is aided by two Mg^2+^ ions interacting with D385 to facilitate contact with the α- and β-phosphates of ATP; this aspartate position is conserved throughout the IP-kinase family. Additionally, H338 can also contribute to charge balance in the catalytic cavity to facilitate γ-phosphate transfer from ATP to InsP3 [41] because of its proximity to the β-phosphate of ATP, one of the magnesium ions, and the 4-phosphate of IP3. Hence, to create a completely kinase-dead IPMK for studies, one may target positions H338 and D385 for mutations to alanine, as well as Q164 for added measure. However, to retain only 3-kinase activity of HsIPMK while completely abrogating 6-kinase activity, one can emulate the double mutant D144N/K146 A [50], though structurally it seems only a K146A mutation should suffice. HsIPMK crystal structures bound to InsP4 or InsP5 were not successful, so the authors of that study [41] modeled in an InsP4 (in silico) into the active site to suggest that InsP3 to InsP5 phosphorylation involves an intermediate dissociation of the InsP4 product followed by rebinding in an orientation achieved by a 180° flip about the 3–6 axis of the inositol ring. However, crystal structures of a different IP6K captured with IP3 and IP5 in its active site [51] suggest an alternative and less energetically costly alternative—a 180° rotation about an axis that is at the center of the inositol ring, such that each inositol carbon position is moved to a new position three numbers down, i.e., 1 to 4, 2 to 5, and so on, in order to position the 6 position in line with the ATP gamma phosphate for transfer.

**Table 2:** IPMK mutational analysis.

Residue	Mutational Effect on 3- or 6- Kinase Activity	Structural Element Involved	Mutants
**Q78**	Both reduced	QPPPR Proline loop, sidechain proximity to Mg^2+^ ion, and b phosphate of ATP	Q78A [42]
**R82**	Both reduced	QPPPR Proline loop, sidechain proximity to 4 and 5 position phosphates of IP3.	R82A [42]
**E131 ^Ψ^**	-	Hinge, backbone proximity to ATP base	No mutational analysis performed for HsIPMK [42]
**V133 ^Ψ^**	-	Hinge, backbone proximity to ATP base	No mutational analysis performed for HsIPMK
**D144**	Only 6-kinase abrogated, 3- kinase activity intact	Proximity to the 3’ hydroxyl of ATP ribose	D144N/K146A double mutant [50]
**K146**	Only 6-kinase abrogated, 3- kinase activity intact	Proximity to IP3 hydroxyl at position 3	D144N/K146A double mutant [50]
**K160**	Both reduced	IP Helices, sidechain proximity to IP3 phosphates at positions 4 and 5	K160 [42]
**Q163**	Both reduced	IP Helices, sidechain proximity to IP3 phosphate at positions 5	Q163A, Q163K, Q163R [42]
**Q164**	Both reduced	IP Helices, sidechain proximity to IP3 hydroxyl at positions 2	Q164A, Q164K, Q164R [42]
**K167**		IP Helices, sidechain proximity to IP3 phosphate at positions 1	K167A [42]
**Q196**		sidechain proximity to IP3 phosphate at positions 1	Q196A, Q196K, Q196R [42]
**D385 ** **^ℵ^**	-	Catalytic site, sidechain proximity to both Mg^2+^ ions and the a and b phosphates of ATP	No mutational analysis performed for HsIPMK [52]
H388 *	Both reduced	Catalytic site, sidechain proximity to a Mg^2+^ ion, and the IP3 phosphate at positions 4	H388A [42,52]

^Ψ^ Conserved residues in ATP binding site similar to Protein Kinase A. ^ℵ,^* Kinase-dead mutations.

While obtaining crystal structures necessitated modifying IPMK protein constructs without the disordered or dynamic regions, these regions have implications for function. Starting with the N-lobe, the disordered region of the ≈60 amino acids influences ATP binding such that its absence manifests in higher catalytic activity. Furthermore, removal of the region that contains the NLS [42] (Figure 2A,B) also results in lower affinity for the InsP3 substrate and perturbs kinetic parameters significantly. Although these perturbations were observed in in vitro experiments, they have implications for IPMK function, as the deleted NLS region is structurally located (Figure 2C) behind the hinge that cradles the ATP base. Interestingly, a putative NES [45] discovered in IPMK is structurally located in the cavity where inositol binds (Figure 2C), insinuating that IPMK enzymatic activity is neutralized during transport out of the nucleus and during interactions with proteins that utilize IPMK sections originating from exons 4 and 5. Overall, while the IPMK crystal structures have provided invaluable information to dissect function, other solution biophysical techniques to interrogate the full-length IPMK could clarify any allostery that may be involved between all these regions. Regardless, crystal structures have been a valuable starting point in the search for IPMK inhibitors and so far have yielded two potential candidates [43]—Quercetin and the antidepressant Vilazodone that target the IPMK ATP binding site.

Lastly, many IPMK–protein interactions (Table 1) have been evaluated based on IPMK fragments whose boundary decisions have been based on exon limits (Figure 2A,C). While these interaction claims have been backed by using the respective fragment/s as a dominant-negative construct, accurately localizing the interaction interface may benefit from designing constructs based on the structure that (a) do not cut into secondary elements and (b) can be confirmed to fold. Of course, these exon boundaries-based fragments may still achieve a small population of folded conformers to act as a dominant-negative construct.

## 4. IPMK-Regulated Cellular Signaling Pathways Influence Various Cell Functions

Research with IPMK mutant cell lines has demonstrated that the loss of IPMK function affects a wide range of cellular mechanisms. These impacts can be attributed to three distinct roles: its function as a lipid kinase, its function as an inositol kinase, or functions that are independent of its kinase activity. The following section will summarize these discoveries.

### 4.1. Cell Functions Require IPMK’s Lipid Kinase Activity

#### 4.1.1. Cell Migration

**Figure 3:**
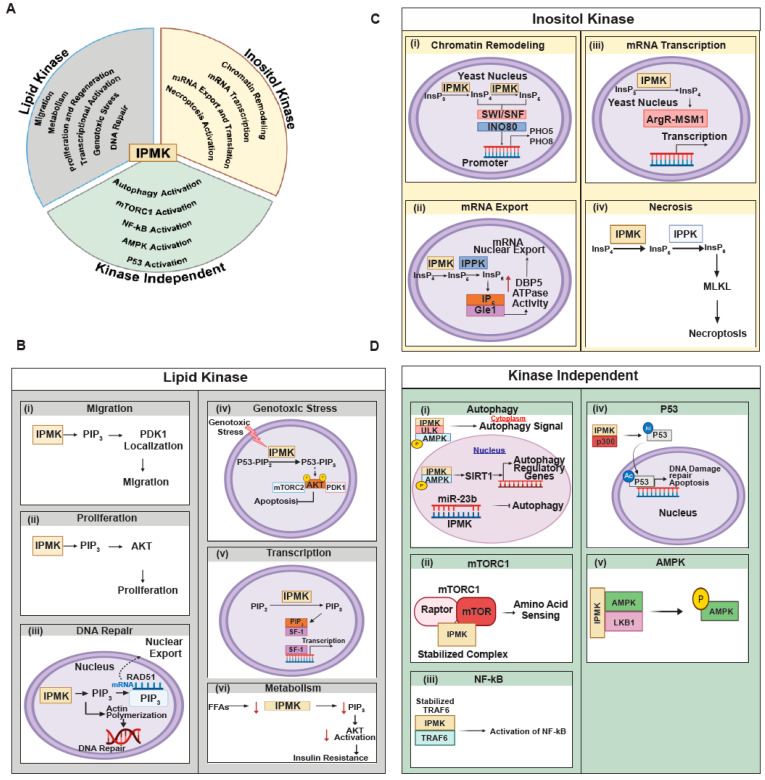
IPMK’s lipid kinase, inositol kinase, and kinase-independent function in the cells. (**A**) IPMK displays several regulatory functions in various cellular pathways using its lipid kinase, inositol kinase, and kinase-independent activities. (**B**) IPMK’s lipid kinase activity influences the following pathways and cellular processes: (**i**) migration [34,53,54,56,57], (**ii**) proliferation [34,58], (**iii**) DNA repair [17,59,60], (**iv**) genotoxic stress [61], (**v**) transcriptional activation [21], and (**vi**) metabolism [58]. (**i**) In migration, IPMK generates PIP_3_, which aids in PDK1 membrane localization and facilitates migration [34]. (**ii**) In proliferation, IPMK generates PIP_3_, which activates AKT and leads to proliferation [34,58]. (**iii**) In DNA repair, IPMK generates PIP_3_, which facilitates actin polymerization in the nucleus, leading to the recruitment of proteins essential for DNA repair [17,59,60]. (**iv**) Genotoxic stress leads to the conversion of p53-PIP_2_ to p53-PIP_3_ by IPMK, which recruits AKT, mTORC2, and PDK1, leading to AKT phosphorylation and activation. The activation of AKT suppresses apoptosis [61]. (**v**) In transcriptional activation, SF-1 binds IPMK-generated PIP_3_, which is essential for SF-1 activation as a transcription factor [21]. (**vi**) In metabolism, free fatty acids (FFAs) decrease IPMK protein levels, leading to a decrease in PIP_3_ and lowering AKT activation. This decrease in AKT activation leads to insulin resistance [58]. (**C**) IPMK’s inositol kinase activity influences the following pathways and cellular processes: (**i**) chromatin remodeling [19,20], (**ii**) mRNA export [62,63], (**iii**) mRNA transcription [22,46], and (**iv**) necroptosis [45,50]. (**i**) In chromatin remodeling, InsP_4_ and/or InsP_5_ generated by IPMK bind to the SWI/SNF and INO80 chromatin remodeling complexes, recruiting them to the promoter sites of yeast PHO5 and PHO8 genes, inducing their transcription [19,20]. (**ii**) In mRNA export and translation, InsP_5_ generated by IPMK is converted to InsP_6_ by IPPK. InsP_6_ binds to Gle1, which allows it to activate the ATPase activity of Dbp5, a protein essential for mRNA nuclear export [62,63]. (**iii**) In mRNA transcription, IPMK-generated InsP_4_ activates Arg-MCM1 transcription factor in yeast, regulating genes involved in cell cycle and cell type specificity [22,46] (**iv**) In necroptosis, InsP_5_ generated by IPMK is converted to InsP_6_ by IPPK. InsP_6_ then binds to MLKL, which activates the necroptotic pathway [50]. (**D**) IPMK’s kinase-independent activity influences the following pathways and cellular processes: (**i**) autophagy [14,64,65,66], (**ii**) mTORC1 activation [23,67,68,69], (**iii**) NF-kB activation [29], (**iv**) p53 activation [48], and (**v**) AMPK activation [30]. (**i**) In autophagy, IPMK, ULK, and AMPK form a complex that leads to AMPK phosphorylation and the activation of the autophagic signal [14,64,65,66]. In the nucleus, IPMK phosphorylates AMPK, which leads to the activation of SIRT1 and the subsequent transcription of autophagy regulatory genes. miR-23b can also bind to IPMK mRNA transcript, suppressing its expression and leading to the inhibition of autophagy. (**ii**) In mTORC1 activation [23,67,69], IPMK stabilizes the mTORC1 complex by binding to raptor and mTOR complex proteins, which leads to the facilitation of amino acid sensing. (**iii**) In NF-kB activation [29], IPMK binds to TRAF6, stabilizing it and leading to the activation of NF-kB. (**iv**) In p53 activation [48], IPMK binds to p300, which leads to the acetylation of p53 and its subsequent DNA recruitment. (**v**) In AMPK activation [30], IPMK binds AMPK and LKB1, leading to the phosphorylation and activation of AMPK.

PIP3 serves as a crucial second messenger in cellular processes, particularly in cell migration [53]. Traditionally, class I phosphoinositide 3-kinase (cPI3K) has been regarded as the primary lipid kinase responsible for the production of PIP3, with the expectation that inhibiting cPI3K would completely halt cell migration. However, studies conducted in the model organism *Dictyostelium*, where cPI3K was genetically eliminated, revealed that cells still migrate but are substantially retarded [53,54]. This finding suggests the involvement of alternative lipid kinases in the generation of PIP3. Recent research has identified IPMK as a potential alternative PI3-kinase that can generate PIP3 independently of cPI3K [34]. Studies by Sekine et al. [54] and Reilly et al. [34] have highlighted the significant role of IPMK’s lipid kinase activity in promoting cell migration in immortalized mouse embryonic fibroblast cells (MEFs). Deletion of *IPMK* or inhibition of its kinase activity resulted in impaired cell migration, indicating its essential function in this process. The precise mechanisms through which IPMK influences cell migration remain to be fully elucidated. One proposed mechanism involves the regulation of integrin expression [54]. Specifically, the loss of IPMK activity may lead to decreased expression of β1 and β3 integrins [54], which are critical cell surface receptors that facilitate cell migration through the activation of focal adhesion kinase (FAK) and Rho signaling pathways. However, attempts to rescue cell migration in IPMK-deleted MEFs by overexpressing β1 integrin were unsuccessful [54], suggesting that integrin-independent pathways likely play a role in *IPMK*-mediated cell migration. In addition to integrin regulation, Reilly et al. [34] proposed an alternative mechanism whereby the loss of IPMK’s kinase activity impairs the membrane localization of 3-phosphoinositide-dependent protein kinase 1 (PDK1) and subsequently affects the phosphorylation of myosin light chain (MLC) by Rho-associated protein kinase 1 (ROCK1). This impairment could hinder cell migration by disrupting actin polymerization. At the molecular level, the interaction between RhoE and ROCK1 inhibits ROCK1’s ability to phosphorylate MLC, thereby preventing cell migration. Upon activation of cell migration, RhoE dissociates from ROCK1, allowing ROCK1 to phosphorylate MLC, which promotes actin polymerization and facilitates cell movement. The localization of PDK1 to the membrane is crucial for the dissociation of the inhibitory RhoE/ROCK1 complex [55]. The loss of IPMK’s lipid kinase activity disrupts PDK1 localization, stabilizing the ROCK1/RhoE inhibitory complex and preventing ROCK1-mediated MLC phosphorylation [55], thus hindering cell migration (Figure 3B-i). In conclusion, while PIP3 has long been recognized for its role in cell migration, emerging evidence points to the involvement of alternative lipid kinases such as IPMK in this process.

IPMK-mediated cell migration promises to yield valuable insights into both disease processes and fundamental cellular mechanisms. Key areas of investigation should include the role of IPMK in cancer metastasis and wound healing, potentially opening new avenues for therapeutic interventions. At the molecular level, elucidating how IPMK’s lipid kinase activity influences major cytosolic signaling pathways such as Rho, Ras, and mTORC2 will be crucial for understanding its impact on cell migration. Furthermore, given IPMK’s unique status as a predominantly nuclear PI3-kinase, exploring its potential to regulate cell migration at the transcriptional level presents an exciting frontier.

#### 4.1.2. Cell Proliferation and Tissue Regeneration

PIP3 plays a crucial role in cell proliferation by activating AKT, a key regulator of cellular growth [70]. Studies by Resnick et al. [26], Maag et al. [15], and Reilly et al. [34] indicated that IPMK is an essential PI3-kinase-generating PIP3, which is critical for AKT activation. Recent research by Reilly et al. [34] further extended the study to evaluate the probable role of IPMK-mediated AKT activation in regulating cell proliferation. The study demonstrated that conditional deletion of *IPMK* in the intestine significantly hinders the spontaneous proliferation of intestinal epithelial cells and AKT signaling. Interestingly, a study by Jung et al. indicated the loss of AKT activation in IPMK-deleted hepatocytes [58]. Though the study did not look for the importance of IPMK in hepatocyte proliferation, there is a possibility of loss of hepatocyte proliferation in *IPMK*-depleted liver due to impaired AKT activation. These findings highlight the essential role of IPMK in maintaining the regenerative capacity of both liver and intestinal tissues.

Future research in liver and intestine regeneration should adopt a comprehensive approach, investigating stem cell activity and the influence of stromal cells on stem cell function. Additionally, future directions should focus on clarifying IPMK’s contribution to cancer cell proliferation, particularly in tumors with aberrant AKT activation. Systematic studies—using genetic and pharmacologic perturbation of IPMK across models with defined PI3K/PTEN/AKT alterations—are needed to determine when IPMK functions as a key enhancer of AKT signaling and growth. These insights would enable rational testing of combination therapies pairing IPMK inhibitors with class I PI3K inhibitors.

#### 4.1.3. IPMK’s Lipid Kinase Activity Influences DNA Repair

Over-expression studies indicate that IPMK is enriched in the nucleus [26], suggesting that PIP3 production also occurs within the nucleus. This nuclear-derived PIP3 is implicated in critical cellular processes such as DNA repair. Two notable studies by Wang et al. [59] and Lamm et al. [60] elucidated that IPMK-generated PIP3 facilitates the polymerization of nuclear actin, essential for the recruitment of ATR (ataxia telangiectasia and Rad3-related protein) and participating in replication stress and DNA repair processes.

A separate investigation by Wickramasinge et al. [61] discovered that IPMK-generated PIP3 in mammalian cells plays a crucial role in regulating the nuclear export of RAD51 mRNA, a key protein in DNA repair. PIP3 recognizes a specific sequence in RAD51 transcripts, facilitating their nuclear export through the ALY factor. While IPMK loss does not affect RAD51 transcription, it impairs the mRNA’s cytoplasmic export and translation, ultimately reducing RAD51 protein production. This finding highlights IPMK’s importance in DNA repair mechanisms beyond its known roles in cell proliferation.

Future research could define IPMK’s role in DNA repair with a particular focus on its precise contribution to double-strand break (DSB) versus single-strand break (SSB) repair. This includes dissecting whether IPMK directly participates in DSB pathways such as non-homologous end joining or homologous recombination, or indirectly influences repair by regulating the expression, localization, or activity of core repair factors. It will also be important to test the hypothesis that IPMK loss drives secondary, stress-mediated mechanisms—such as oxidative or proteostasis stress—that impair repair capacity and thereby promote DNA damage.

#### 4.1.4. PI3-Kinase Activity of IPMK Regulates Genotoxic Stress-Mediated Cell Death

While years of research have primarily investigated AKT signaling at the cell membrane, recent works by Chen et al. [71] and Choi et al. [61] have revealed that, under genotoxic stress, IPMK-generated nuclear PIP3 binds to p53, which activates nuclear AKT signaling and suppresses apoptosis. This finding highlights a membrane-independent mechanism where IPMK acts as a nuclear PI3K, forming p53-PIP3 complexes that recruit and activate AKT within the nucleus, representing a new dimension in cell survival regulation.

Future research could aim to elucidate how nuclear PIP3 regulates nuclear functions of proteins traditionally known for cytoplasmic roles, such as mTORC1 and mTORC2, which are increasingly recognized to possess critical nuclear activities, including gene regulation, chromatin remodeling, and DNA repair. Investigating the mechanisms by which nuclear PIP3 modulates the localization, activation, and functional integration of these and similar proteins will open new avenues for understanding nuclear signaling networks and may uncover novel therapeutic strategies for diseases where these pathways are dysregulated.

#### 4.1.5. IPMK’s PI3-Kinase Activity Influences Transcription

IPMK’s PI3K activity is crucial for activating Steroidogenic factor 1 (SF-1)-mediated transcription [21]. SF-1 is a key transcription factor in endocrine networks, regulating genes essential for steroidogenesis, sexual development, and reproduction. IPMK converts PIP2 to PIP3, which is necessary for SF-1-mediated transcriptional activation (Figure 3B). *IPKM* knockdown or mutations affecting PIP2 binding to SF-1 disrupt this process.

### 4.2. Cell Function That Requires IPMK’s Inositol Kinase Activity

IPMK is a rate-limiting enzyme in the Higher Order Inositol Phosphate (HOIP) pathway, significantly impacting cellular inositol phosphate levels [14]. When *IPMK* is deleted, InsP5 and InsP6 concentrations within cells show a notable reduction, highlighting its importance in maintaining these HOIPs [14]. This section will discuss cellular functions and molecular mechanisms that are regulated by IPMKs’ inositol kinase activity.

#### 4.2.1. Chromatin Organization

The pivotal role of IPMK in regulating chromatin remodeling and transcription was first discovered by Steger et al. in yeast [19]. In their study, depletion of *IPMK*, also known as IPK2 or Arg82, hindered the recruitment of SWI/SNF and INO80 chromatin remodeling complexes to the promoters of phosphate-responsive genes such as PHO5 and PHO8 (Figure 3C). Mutational analysis revealed that InsP4 and/or InsP5, generated by IPK2, are crucial for modulating SWI/SNF and INO80 complexes’ ability to induce transcription of phosphate-responsive genes [19]. Recent research by Beon et al. [20] expanded on these findings, showing that IPMK physically interacts with the mammalian SWI/SNF complex by directly binding to SWI/SNF complex components SMARCB1, BRG1, and SMARCC1. This interaction subsequently facilitates chromatin remodeling and transcriptional regulation in mammalian cells. Importantly, Beon et al. [20] found that, unlike in yeast, where inositol phosphate is required for chromatin remodeling, the effect of IPMK on chromatin remodeling in mammalian cells is kinase-independent.

The SWI/SNF complex is indeed a crucial player in chromatin remodeling, and its dysregulation is linked to cancer. The SWI/SNF complex interacts directly with key tumor suppressors and oncogenes such as RB, BRCA1, c-MYC, and MLL, impacting genes crucial for cell proliferation, differentiation, and survival [72]. The precise relationship between IPMK and the activation of the SWI/SNF complex in cancer and other diseases remains to be elucidated.

#### 4.2.2. mRNA Transcription in Yeast

The York group demonstrated that InsP4 (Ins 1, 4, 5, 6) is crucial for activating the ArgR-MCM1 transcription factor in yeast [73], which regulates genes involved in cell cycle, cell type specificity, and responses to pheromones and nutrients. This study revealed that IPK2 (yeast IPMK) enhances DNA binding of the ArgR-MCM1 complex independently of its kinase activity. However, the transcriptional function of this complex is regulated by InsP4 and InsP5, which are products of IPK2’s kinase activity. In mammalian cells, the ortholog of MCM1, Serum Response Factor (SRF), activates immediate early genes [72,74]. Kim et al. [46] found that mammalian IPMK acts as a transcriptional co-activator of SRF, promoting gene transcription through physical interaction with SRF, independent of its kinase activity.

#### 4.2.3. mRNA Export and Translation

The Wente’s lab discovered that InsP6 plays a crucial role in nuclear mRNA export and translational termination by interacting with the Gle1 protein in yeast [62]. InsP6 binding to Gle1 enables it to stimulate the ATPase activity of Dbp5, a DEAD-box protein essential for mRNA export. While Gle1’s interaction with InsP6 is critical for mRNA export, later studies from the same group found the role of InsP6/Gle1 complex in efficient translational termination [63]; however, Gle1 can regulate translation initiation independently of InsP6 [63]. The impact of IPMK’s inositol kinase activity, specifically its loss, on mRNA metabolism and transport in mammalian systems remains unresolved.

#### 4.2.4. Necroptosis

Necroptosis is a specialized lytic cell death pathway [50] often triggered by inflammatory conditions independent of other classical cell death pathways like apoptosis, autophagy, pyroptosis, and ferroptosis. TNF alpha, a classical inflammatory cytokine, triggers necroptosis by activating RIPK3 (Receptor Interacting Protein Kinase 3) and MLKL (Mixed Lineage Kinase Domain-Like) [50]. Through a blind genome-scale knockout screening, Dovey et al. [50] identified IPMK as an important regulator of necroptosis. Mechanistically, InsP6 binds to MLKL, displacing auto-inhibitory braces and activating the necroptosis pathway.

Future research could explore the clinical implications of this finding, as necroptosis causes brain cell death during a stroke [75]. Regulating InsP6 levels may be a therapeutic option for brain stroke or other inflammatory conditions related to necroptotic cell death. Additionally, it would be interesting to explore how IPMK may influence other cell death pathways, such as pyroptosis, ferroptosis, apoptosis, etc., which have not been explored yet.

#### 4.2.5. IPMK’s Inositol Kinase Activity Regulates Histone Acetylation in Mammalian Cells

The first evidence that HOIPs bind to histone deacetylase 3 (HDAC3) came from crystal structure analyses, which revealed InsP4 acting as a molecular “glue” between HDAC3 and the deacetylase activator domain (DAD) of its coregulator protein SMRT [76]. Later studies suggested that higher-order inositol phosphates (such as InsP6) might also interact with HDAC1 [77]. However, these early findings did not address how the loss of HOIPs would affect the physiological activation of HDAC3 or HDAC1, nor the consequences for gene expression.

Recent research has shown that depletion of IPMK—responsible for synthesizing HOIPs—in cells or mouse tissue significantly diminishes HDAC3 activation and results in increased histone acetylation [78,79]. Additionally, disease-relevant connections have been identified: in both mouse models and patient tissues of age-related macular degeneration (AMD) [80], reduced levels of IPMK result in impaired HDAC3 activation, disrupted gene regulation, and increased proteostatic stress.

What remains unclear is whether IPMK loss specifically impairs HDAC3 activation or also affects HDAC1 and other histone deacetylases, such as sirtuins.

### 4.3. IPMK Influences Cell Function in a Kinase-Independent Manner

IPMK, despite its PI3-kinase and inositol kinase activities, influences various cellular functions through kinase-independent mechanisms, primarily via protein–protein interaction. Here are some examples (Figure 3B).

#### 4.3.1. Autophagy

Autophagy functions as a cellular quality control system, targeting and eliminating misfolded proteins, protein aggregates, and damaged organelles such as mitochondria [81]. *IPMK* depletion impairs autophagy activation in both yeast [64,65] and mammalian cells [14,82]. Studies by Guha et al. [14,82] revealed that IPMK activates the autophagy regulator pathway at both cytoplasmic and nuclear levels. In the cytoplasm, IPMK acts as a scaffold protein, forming an IPMK/ULK/AMPK ternary complex that activates the autophagic signal. In the nucleus, IPMK binds to AMPK, which activates Sirtuin-1 (histone deacetylase), leading to the transcription of autophagy regulatory genes. However, Chen et al. [49] found the opposite effect in *C. elegans,* where IPMK negatively controls autophagy by deactivating TFEB.

Recent studies by Pan et al. [83] and Hu et al. [84] have identified IPMK as an autophagy activator, supporting Guha et al.’s findings. Interestingly, Pan et al. [83] demonstrated that loss of *IPMK* impairs autophagy activation in osteosarcoma cells when treated with Ebastine, a second-generation antihistaminic drug effective in inhibiting osteosarcoma cell proliferation. The study revealed that Ebastine activates IPMK-mediated AMPK/ULK activation, inducing autophagy in these cells. Knocking down *IPMK* reduced Ebastine-induced autophagy. Separately, Hu et al. [84] identified that microRNA-23b (miR-23b) directly binds to IPMK and represses its expression, thereby inhibiting autophagy. miR-23b was downregulated in intracerebral hemorrhage (ICH), leading to increased *IPMK* expression and autophagy-related toxicity. Overexpressing miR-23b alleviated ICH pathology, reduced *IPMK* expression, and diminished autophagy. These studies indicate a cell and organism-specific role of IPMK in activating autophagy.

Future research is needed to evaluate the importance of IPMK in regulating selective autophagy, such as mitophagy and ribophagy, and to understand how IPMK maintains autophagic flux, which is critical for cell homeostasis. Investigating IPMK’s role in circumstances where autophagy is dysregulated, like impaired liver regeneration due to loss of autophagy, drug-induced hyperactivation of autophagy, as in cocaine abuse-induced autophagic cell death in the brain [66], could provide valuable insights into the underlying mechanisms.

#### 4.3.2. mTORC1 Activation and Nutrient Sensing

mTORC1 signaling is a major nutrient-sensing pathway that regulates translation. Kim et al. [23] were the first to demonstrate that IPMK stabilizes the mTORC1 complex and facilitates amino acid sensing in cells. This function of IPMK in regulating mTORC1 activation does not require its kinase activity; instead, IPMK directly binds to mTORC1 complex components—mTOR and Raptor, thereby stabilizing the complex. Recent cryo-electron microscopy studies have elucidated the structures of mTORC1 and mTORC2, identifying a conserved InsP6 binding site in mTOR [67,68]. Furthermore, Rameh et al.’s in vitro study suggests the role of InsP6 and other HOIPs in the positive regulation of mTOR’s catalytic activity [69]. However, the role of InsP6 in either activating or inhibiting mTORC1/2 remains unclear.

#### 4.3.3. AMPK Activation

*IPMK* depletion impairs AMPK activation [30], which is crucial for glucose sensing and metabolism. Studies have shown that deletion of *IPMK* reduces LKB1-mediated AMPK phosphorylation, resulting in AMPK deactivation. IPMK directly binds to AMPK and its upstream kinase, suggesting the formation of a complex involved in AMPK activation. However, it is unclear whether the binding alone is sufficient for AMPK’s kinase activity or if IPMK’s kinase activity also plays a role.

#### 4.3.4. p53 Activation

Genotoxic stress activates p53-mediated transcription of genes involved in apoptosis and DNA damage/repair [85]. Xu et al. [48] demonstrated that *IPMK* loss significantly impairs p53-mediated transcription in cell lines. Mechanistically, IPMK directly binds to the protein acetylase p300, facilitating p53 acetylation, which is critical for its stability and DNA recruitment. This suggests that IPMK may contribute to the activation of p53.

#### 4.3.5. NF-kB Mediated Transcription

Toll-like receptor activation plays a central role in inflammatory signaling, activating NF-kB (nuclear factor kappa beta) transcription factors and subsequent transcriptional activation of inflammatory genes [29]. TRAF6 (Tumor necrosis factor receptor-associated factor 6) is essential for translating Toll-like receptor-mediated signals into NF-kB activation [29]. IPMK binds to TRAF6 and stabilizes it by impeding its proteasomal degradation, thereby activating NF-kB downstream [29].

#### 4.3.6. Role of IPMK in YAP Signaling

Recent findings by Jung et al. 2024 [86] reveal that nuclear phosphoinositides act as critical cofactors facilitating the interaction between YAP/TAZ and TEADs. Specifically, the enzymatic products of phosphoinositide kinases PIPKIα and inositol polyphosphate multikinase (IPMK)—namely PI(4,5)P2 and PI(3,4,5)P3—serve as molecular bridges that stabilize YAP/TAZ binding to TEAD [86]. Disruption of these lipid signals, either by inhibiting PIPKIα/IPMK activity or blocking YAP/TAZ interaction with these phosphoinositides, significantly reduces YAP/TAZ-TEAD binding, suppresses downstream target gene expression, and impairs breast cancer cell motility. While the potential role of other IPMK products, like inositol phosphates, cannot be completely ruled out [82].

#### 4.3.7. Role of IPMK in DNA Methylation

A recent study revealed that IPMK is a key regulator of DNA methylation [87], as its depletion in cells led to changes in over 22,000 differentially methylated regions (DMRs). By integrating data from DMR-affected genes and RNA-seq, the study identified 35 genes showing an inverse correlation between promoter methylation and gene expression, with pathway analysis indicating impacts on tissue remodeling and hematopoiesis. Notably, loss of IPMK resulted in significant promoter methylation changes and decreased mRNA and protein levels for MMP14 and LIF [87]. These findings position IPMK as a novel regulator of DNA methylation.

## 5. Importance of IPMK in Physiology and Diseases

**Figure 4:**
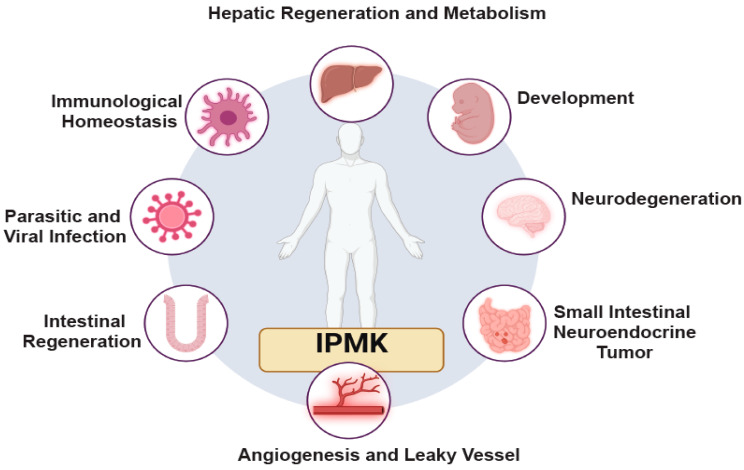
Role of IPMK in influencing physiology and disease. IPMK plays a role in several diseases and physiological processes, including hepatic regeneration and metabolism [14,58,88], development [24,25,89], neurodegeneration [90,91], small intestinal neuroendocrine tumor [35], angiogenesis and leaky vessels [13], intestinal regeneration [33,34], parasitic and viral infection [92,93,94,95], and immunological homeostasis [28,96,97].

While cell biology studies have established IPMK’s role in regulating diverse cellular mechanisms, evidence from preclinical models and human genome-wide association studies also implicates IPMK in human disease. These studies connect alterations in IPMK—such as loss-of-function, gain-of-function, and other mutations—to various conditions. The following section explores these disease-related findings (Figure 4).

### 5.1. Developmental Abnormalities and Embryonic Lethality

Studies involving whole-body deletion of *IPMK* have identified severe developmental defects in both mice and *Drosophila melanogaster* (*fruit fly*).

#### 5.1.1. Mice

The importance of HOIPs, specifically InsP4-6, became evident with the discovery that mice with specific *IPMK* deletions were embryonically lethal [25]. Homozygous *IPMK* null mutants displayed developmental delays and were smaller than normal embryos and showed embryonic lethality by 9.5–10 embryonic days [25]. Pathological and histological analysis revealed several abnormalities, such as the failure of allantois fusion with the chorion, absence of somites, and significant folding defects and kinking of the neural tube, particularly in the mid-hind region of the embryo. During development, specific signaling gradients are enriched in selective parts of the embryo, impacting organ development. John Verbsky et al. [89] identified IPMK expression in E9 embryos using a β-galactosidase reporter mouse model created in their lab. Robust IPMK staining was observed in neural tubes, notochords, and somites, underscoring the IPMK’s presence in developing somites and neural tubes. Hence, these two key studies have established IPMK’s critical role in mouse development, specifically in the formation of the neural tube and the placenta. While the mechanism behind IPMK’s function in neural tube development remains unexplored, the impaired placental development observed in its absence initially suggested that IPMK loss might suppress angiogenesis (the formation of new blood vessels). However, subsequent research by Fu et al. [13] demonstrated the opposite: IPMK loss in cell lines and the mouse brain actually induces angiogenesis, pointing to a more complex regulatory function. This paradoxical finding raises the hypothesis that IPMK loss may lead to uncontrolled or “leaky” vessel development, a phenomenon also seen during the over-activation of DLL4-mediated Notch signaling.

Future research could clarify how IPMK regulates angiogenesis, especially whether its loss results in abnormal or leaky vessels that affect placental development through dysregulated Notch signaling. Additionally, investigating the mechanisms by which IPMK influences neural tube development—an area that remains unexplored—could reveal new IPMK target pathways. Since genes involved in development are often dysregulated in cancer, uncovering these mechanisms could also provide insights into the largely unknown role of IPMK in tumorigenesis.

#### 5.1.2. Drosophila Melanogaster (Fruit Fly)

Deletion of *IPMK* in *Drosophila* larvae reduced InsP5 and InsP6 production and resulted in severe apoptosis in imaginal disks [24]. Interestingly, the author observed that depletion of IPMK led to apoptosis in the imaginal disk, as well as reduced cell proliferation in the wing disk. While a proper balance between apoptosis and cell proliferation is generally crucial for developmental success, the pathways regulating these two processes are often distinct. The author attempted to identify the mechanism underlying apoptosis by testing dominant-negative forms of JNK and p53, but these experiments did not yield conclusive results. Instead, the study found that loss of the JAK-STAT pathway may be responsible for the observed reduction in cell proliferation following IPMK depletion. Furthermore, the authors suggested that the inositol kinase activity of IPMK is likely important for JAK-STAT pathway activation in Drosophila. Since Drosophila is a crucial model system, studies in this organism have often helped identify parallel mechanisms in mammalian and human cells. In this context, identifying IPMK as a critical developmental gene—whose loss increases apoptosis and decreases cell proliferation—suggests that a loss of IPMK function in mammalian systems could result in a similar phenotype.

Given these findings, future studies could investigate whether the loss of IPMK function results in comparable effects on apoptosis and cell proliferation in human cells. Determining whether IPMKs’ inositol kinase activity similarly regulates the JAK-STAT pathway in these systems could provide valuable insights into its broader biological role. Additionally, exploring the specific molecular mechanisms by which IPMK influences pathway activation may uncover new therapeutic targets for diseases associated with abnormal cell proliferation and apoptosis.

### 5.2. Intestinal Diseases

IPMK dysfunction has been directly linked to human intestinal diseases, with genome-wide association studies strongly connecting *IPMK* to inflammatory bowel disease (IBD), which includes Crohn’s disease and ulcerative colitis. Independent studies by Reilly et al. [34] and Park et al. [33] have demonstrated that *IPMK* deletion in mouse intestinal epithelial cells significantly impairs regeneration and aggravates inflammation. While GWAS and experimental studies implicate IPMK in intestinal function, its precise roles in inflammation, barrier integrity, stem and specialized epithelial cells, and the microbiome remain unclear. Notably, recent findings on IPMK’s impact on gut permeability in intestine-specific knockout mice are contrasting: Park et al. [33] observed no alteration in intestinal permeability, whereas Chatterjee et al. [78] reported a significant increase following IPMK deletion. This discrepancy may be attributed to differences in the genetic background of the mouse models. Park et al. used IPMK-floxed mice on a mixed 129SV–C57BL/6 background with only five generations of backcrossing, potentially retaining residual donor alleles. In contrast, Chatterjee et al. used mice on the same background, but backcrossed for ten generations, which is the gold standard [98] for reducing genetic variability. Incomplete backcrossing can leave background alleles that influence intestinal phenotypes—potentially compounded by alterations in the gut microbiome—which may account for the divergent results.

Considering that increased gut permeability is an early hallmark of Inflammatory Bowel Disease (IBD), future studies are essential to definitively clarify IPMK’s role in intestinal biology and barrier function. Key research could investigate the specific mechanisms involved, including how *IPMK* deletion impacts the diversity of the gut microbiome and its influence on specialized epithelial cells, such as stem, paneth, enteroendocrine, and goblet cells. Furthermore, it is critical to determine if the loss of IPMK function leads to chronic intestinal inflammation and whether this elevates the risk of intestinal cancer in older mice. Addressing these questions will enhance our understanding of how IPMK sustains intestinal homeostasis and how its dysfunction contributes to disease progression.

### 5.3. Intestinal Neuroendocrine Tumor

The discovery of genetic alterations in the *IPMK* gene in familial small-intestine neuroendocrine tumors (SI-NETs) has provided insights into IPMK’s potential role in the pathogenesis of these rare cancers. Wank and colleagues [35] identified a germline 4 bp deletion in *IPMK* in affected families, leading to reduced kinase activity and nuclear localization of the IPMK protein. This mutation resulted in decreased InsP5 formation and impaired p53 activation, affecting apoptosis and DNA damage response. However, subsequent studies failed to find *IPMK* mutations in additional familial SI-NET patients [99], suggesting genetic heterogeneity in the disease. These conflicting findings underscore the complexity of SI-NETs’ genetic landscape and indicate that IPMK mutations may not be a universal driver of familial cases.

Future research is necessary to fully understand IPMK’s role in SI-NET pathogenesis. Engineering SI-NET–specific IPMK mutations in mice would be an effective approach to clarify the role of IPMK in the development and progression of small intestinal neuroendocrine tumors (SI-NET). Additionally, since human data suggest a role for IPMK in intestinal tumors, it would be valuable to investigate the effects of IPMK mutations, loss, or gain of function in both human tissue and murine models of colon cancer, which is more prevalent.

### 5.4. Liver Regeneration and Insulin Signaling

Liver regeneration is essential for liver wound healing, and Guha et al. [14] observed the critical role of IPMK in this process. Their research showed that hepatocyte-specific *IPMK* knockout mice experienced impaired liver regeneration due to defective lipophagy. Recent studies also revealed that IPMK is vital for regulating hepatic insulin signaling and gluconeogenesis [88], both in vitro and in vivo. *IPMK*-deficient hepatocytes exhibited impaired insulin-induced activation of the AKT-FoxO1 signaling pathway and elevated levels of gluconeogenic enzymes, issues that could be reversed by treatment with SC79, a specific Akt activator. Additionally, in mice fed a high-fat diet, the deletion of *IPMK* in hepatocytes exacerbated hyperglycemia and insulin sensitivity [58]. These mice showed increased hepatic glucose production during a pyruvate tolerance test and decreased Akt phosphorylation in the liver. The loss of hepatic IPMK led to reduced insulin-stimulated AKT phosphorylation and downstream signaling, resulting in increased glucose synthesis and worsened insulin resistance. These findings underscore the importance of IPMK in mediating insulin signaling and gluconeogenesis, highlighting its potential as a therapeutic target for type 2 diabetes.

### 5.5. Immune Signaling

#### 5.5.1. T-Cell

A recent study has shed light on the crucial role of IPMK in the activation of regulatory T-cells (Tregs) [100]. Researchers developed Treg-specific *IPMK* knockout mice and demonstrated that IPMK is essential for the generation and maintenance of RORγt+ Treg cells and their regulatory functions. Tregs play a vital role in preventing autoimmune disorders by inactivating self-reactive T-cells, and impaired Treg function can lead to tissue inflammation. The study showed that *IPMK* deletion impaired Treg function, resulting in intestinal inflammation in mice. In the tumor microenvironment, Treg recruitment typically suppresses effector T-cell-mediated immune responses, but the loss of Treg’s anti-inflammatory properties enhances effector T-cell function. Min et al. [100] demonstrated that *IPMK*-deleted Treg mice exhibited enhanced antitumor efficacy of effector T-cells and significantly reduced melanoma size compared to wild-type mice. These results were supported by RNA-seq analysis, which revealed that *IPMK* loss impaired Treg function by impacting Treg-specific transcriptional programs. The expression of transcription factors such as Ahr, Batf, Maf, and Prdm1, which facilitate Treg cell differentiation and function, was downregulated in *IPMK*-deficient Treg cells. Conversely, Tcf7 and Satb1, which are associated with the naive state, were upregulated. These findings highlight the critical role of IPMK in regulating Treg function and suggest its potential as a target for modulating immune responses in autoimmune disorders and cancer therapy.

#### 5.5.2. T Helper Cells

A study by Hong et al. (2024) [101] showed that CD4-specific IPMK knockout mice have shown a marked reduction in PLCγ1 Y783 phosphorylation in CD4+ helper T-cells, leading to diminished calcium signaling and decreased IL-2 production. The study revealed that IPMK facilitates PLCγ1 activation by directly binding to Sam68 (Src-associated substrate during mitosis of 68 kDa). Mechanistically, IPMK stabilizes the interaction between Sam68 and PLCγ1, thereby enhancing PLCγ1 phosphorylation. Disrupting the IPMK–Sam68 interaction with dominant-negative IPMK peptides led to reduced PLCγ1 phosphorylation, underscoring the critical role of this protein–protein interaction in regulating PLCγ1 activation.

A recent study by Zou et al. (2024) [97] revealed through single-cell analyses of CD4+ T-cells from asthma and chronic rhinosinusitis patients that Th2 cells exhibit elevated expression of hypoxia-inducible factor 2α (HIF2α), which plays a crucial role in their pathogenic differentiation. Using single-cell and lineage tracing methods in HIF2α-deficient mice, they identified a differentiation pathway from TCF1+Ly108+ stem-like Th2 cells to ST2+CD25+ pathogenic Th2 cells, regulated by a HIF2α-GATA3 transcriptional circuit. A key component of this process is the upregulation of inositol polyphosphate multikinase (IPMK), which modulates phospholipid metabolism and enhances T-cell receptor (TCR)-PI3K-AKT signaling. Importantly, overexpression of IPMK in HIF2α-deficient cells restored PIP3 synthesis and rescued pathogenic Th2 differentiation, while pharmacological inhibition of HIF2α led to decreased IPMK expression, impaired Th2 cell pathogenicity, and reduced airway inflammation. These findings highlight IPMK as a critical downstream effector of HIF2α and a promising therapeutic target for controlling Th2-mediated inflammation in asthma.

A recent study by Yuk et al. (2025) [102] described the role of inositol polyphosphate multikinase (IPMK) in Th1 and Th17 cell differentiation. CD4-specific deletion of *IPMK* in T-cells results in weakened Th1- and Th17-mediated immune responses, leading to reduced resistance against *Leishmania major* infection and alleviation of experimental autoimmune encephalomyelitis. These IPMK-deficient CD4+ T-cells exhibit impaired activation and Th17 differentiation, associated with decreased activation of the Akt, mTOR, and STAT3 signaling pathways. Mechanistically, IPMK acts as a phosphatidylinositol 3-kinase, facilitating the production of phosphatidylinositol (3,4,5)-trisphosphate (PtdIns (3,4,5) P3), which is essential for effective T-cell activation and effector functions. Notably, in the absence of IPMK, TCR-stimulated PtdIns (3,4,5) P3 generation is abolished by wortmannin treatment, indicating that IPMK operates in a wortmannin-sensitive manner.

#### 5.5.3. B-Cell

A study employing B-cell-specific *IPMK* knockout mice has unveiled the significance of HOIP (Higher Order Inositol Pathway) in T-cell-independent B-cell activation [96]. In mammals, B-cell-mediated humoral immunity is crucial for defense against microbial infections [103]. The research demonstrated that the IPMK-mediated HOIP pathway regulates B-cell proliferation, with *IPMK* deletion resulting in reduced B-cell numbers [96]. Mechanistically, the study revealed that in the B-cell, InsP5/6 (inositol pentakisphosphate and inositol hexakisphosphate) activates Bruton’s tyrosine kinase, which, in turn, activates the B-cell receptor and promotes B-cell proliferation. Notably, the researchers were able to restore B-cell activation and proliferation in *IPMK*-deleted B-cells by supplementing them with cell-permeable InsP6. This finding underscores the critical role of IPMK-mediated HOIP signaling and suggests a potential therapeutic target for modulating humoral immune responses.

#### 5.5.4. Macrophage-Mediated Immune Response

Studies by Kim et al. [29] and Ahn et al. [28] underscore the significance of IPMK in regulating innate immune responses mediated by myeloid cells, particularly macrophages. Kim et al. [29] found that the loss of *IPMK* protected mice from lipopolysaccharide (LPS)-induced inflammation by downregulating toll-like receptor activation, suggesting that *IPMK* deletion may attenuate inflammatory responses triggered by bacterial endotoxins. In contrast, Ahn et al. [28] discovered that *IPMK* deletion did not reduce inflammation in mice challenged with intraperitoneal K/BxN (inflammatory arthritis mouse model) serum injection to induce arthritis-like symptoms. Instead, they observed an increase in arthritis scores in *IPMK*-deficient mice, indicating a potential exacerbation of inflammatory responses in this context. These findings highlight the complexity of IPMK’s role in modulating macrophage-mediated immune responses and suggest that its effects are context-dependent.

Given the immune system’s complexity and its central role in both tissue inflammation and cancer therapies, understanding how IPMK regulates immune cell activation within diverse tissue contexts is critical for clarifying disease mechanisms. Future studies should focus on elucidating the specific pathways through which IPMK promotes or suppresses immune functions—such as its roles in modulating T and B cell signaling, cytokine production, and inflammatory responses—in the context of particular disease systems. Additionally, investigating how HOIP influences immune cell activation and function represents another compelling research direction, given its established impact on inflammation, cell death pathways, and anti-tumor immunity.

### 5.6. Leaky Vessel

Fu et al. [13] investigated IPMK’s role in vessel formation and angiogenesis, with a focus on brain vasculature integrity. Their findings revealed that *IPMK* deletion in stromal cells, such as mouse embryonic fibroblasts (MEFs), led to increased secretion of pro-angiogenic factors like VEGF, resulting in enhanced vessel formation through HIF1alpha hyperactivation. Notably, when *IPMK* was specifically deleted in brain cells using Nestin Cre driver mice, a significantly higher vascular density was observed in the cerebral cortex. However, this increase in vessel density was accompanied by compromised vessel integrity, leading to a leaky blood–brain barrier. These results suggest that while IPMK may play a role in regulating vessel formation and angiogenesis, its loss could potentially result in impaired vascular integrity, particularly in the brain.

Interestingly, IPMK depletion produces an angiogenic phenotype that closely parallels the effects seen with loss of the NOTCH ligand DLL4 in blood vessels, where DLL4 deficiency leads to aberrant and leaky vessel formation [104]. This similarity suggests a potential connection between IPMK and DLL4-mediated NOTCH signaling in regulating vascular integrity and patterning. This suggests a possible functional link between IPMK and DLL4-mediated NOTCH signaling, raising the intriguing possibility that IPMK may influence angiogenesis through this pathway. Future studies exploring the mechanistic relationship between IPMK and NOTCH signaling and their cross-talk under hypoxia could provide valuable insight into its role in vascular development.

### 5.7. Parasitic and Viral Infections

The emerging role of the HOIP pathway, particularly involving IPMK, as a potential drug target for combating parasitic infections such as *Trypanosoma brucei* and HIV is promising. Studies [92,93] have shown that inhibiting IPMK in *T. brucei* (TbIPMK) can protect mice from infection, and chemical inhibitors targeting TbIPMK have been identified, showing efficacy in killing intracellular parasites.

Additionally, research indicates that InsP6 is crucial for HIV particle assembly [94,95], and inhibitors of IPMK could potentially hinder HIV propagation.

### 5.8. Neurodegeneration

#### 5.8.1. Huntington’s Disease

Studies by Ahmed et al. [90] demonstrated a significant loss of IPMK in the striatal brain samples of both humans and mice with Huntington’s disease (HD), suggesting IPMK’s involvement in the pathogenesis of this neurodegenerative disorder. The study also proposed a potential therapeutic intervention for HD by showing that rescuing IPMK expression in the brains of HD mice via adenoviral transfection reduced mutant HD aggregates and improved locomotor activity.

#### 5.8.2. Alzheimer’s Disease

A 2016 study explored the genetic overlap between Alzheimer’s disease (AD) and immune-mediated diseases [32], revealing a significant association between the SNP rs12570088 at the *IPMK* locus and neurofibrillary tangle (NFT) pathology in AD. Interestingly, this SNP was also linked to Crohn’s disease in the intestine, suggesting a potential connection between gut inflammation and AD. The study confirmed lower IPMK expression in postmortem AD samples, indicating that dysregulation of IPMK expression or function may contribute to AD development.

Another study investigating the relationship between Late Onset Alzheimer’s Disease (LOAD) and longevity found that certain IPMK SNPs, previously associated with decreased longevity, were protective factors for LOAD [91]. This finding underscores the complexity of genetic factors influencing disease risk and longevity, suggesting that genes affecting longevity may also play a role in the development of age-related diseases like AD.

Future research investigating the roles of IPMK and HOIPs in Alzheimer’s disease (AD) pathology and the aging brain may open new avenues for understanding and potentially treating this devastating neurodegenerative disorder. Utilizing mouse models of neurodegenerative diseases to assess IPMK expression, PIP3 levels, or HOIP abundance could yield valuable insights. Rescue experiments—such as cranial injection of IPMK or treatment with InsP6—might also uncover novel therapeutic strategies. Importantly, future research should focus on elucidating how IPMK influences brain function and related biochemical pathways in specific neuronal cell types, including neurons and glia, as this remains an unexplored but promising direction for identifying new mechanisms in neurodegeneration.

## 6. Discussion

Research over the past two decades has revealed IPMK as a multifaceted enzyme with diverse functions spanning from the nucleus to the cytoplasm. It plays a crucial role in cell proliferation [34], metabolism [58], autophagy [14,64], and migration [34,53,54]. Studies involving *IPMK* gene disruption in mouse models and human mutation analyses have highlighted its potential involvement in various diseases.

IPMK’s molecular function is intriguing due to its versatile modes of action. This enzyme can modulate cellular signaling through multiple mechanisms: by generating highly phosphorylated inositol phosphates (HOIPs) [10,12,14], producing phosphatidylinositol 3,4,5-trisphosphate (PIP3) [10,12,14], or even functioning independently of its kinase activity through protein–protein interaction [10,11,12,14].

A critical yet underexplored aspect of IPMK is its precise intracellular distribution, which is essential for understanding its cellular function. Meyer et al. [45] were the first to investigate this in-depth, using immunofluorescence analysis of overexpressed N-terminal EYFP-IPMK fusion constructs. Although they also mentioned cell fractionation studies, the data was not presented. Their work with epifluorescence microscopy suggested that IPMK is distributed in both the nucleus and the cytoplasm. Consistent with this, Meyer et al. [45] identified a nuclear export signal (NES, amino acid 170–179) in addition to IPMK’s known C-terminal nuclear localization signal (NLS), suggesting that IPMK may shuttle between these compartments. To validate this compelling hypothesis, further research is required. This includes confocal imaging of fixed and live cells, along with cell fractionation studies, to observe endogenous IPMK. Creating NLS and NES mutants would also be crucial to map their distribution and determine how these mutations influence cellular mechanisms. In contrast to overexpression systems, tracking endogenous IPMK is the ideal approach. However, a major obstacle is the lack of reliable antibodies, as currently available commercial and lab-generated antibodies produce multiple nonspecific bands in Western blots and have not been validated for immunofluorescence. Therefore, generating a validated monoclonal antibody is a priority for accurately detecting endogenous IPMK localization. Ultimately, this would allow researchers to determine how IPMK activates specific signaling pathways in response to stimuli and to map the subcellular regions where these events occur. For example, while IPMK is known to activate mTORC1, it remains unclear whether this activation occurs on the lysosome, in the cytoplasm, or within the nucleus, where mTORC1 has recently been found. Pinpointing the location of this interaction is key to understanding its influence on cell function.

Overall, it indicates that despite extensive research on IPMK’s diverse roles in cell signaling, function, and physiology, a significant knowledge gap persists regarding its primary regulatory pathway in maintaining cellular homeostasis. It remains unclear which specific physiological or cellular functions are principally regulated by IPMK. Identifying these key processes is crucial for fully understanding IPMK’s importance in cellular biology and for potentially leveraging it as a therapeutic target.

## 7. Conclusions

IPMK is a distinctive protein that serves both as an inositol kinase and a lipid kinase, and its enrichment in the nucleus highlights a novel area for exploring the roles of HOIP and PIP3 in nuclear function, a field that remains largely uncharted. Genome-wide association studies (GWAS) have identified IPMK as a risk gene for inflammatory bowel disease (IBD), prompting investigations into its function through the lens of inositol and PIP3-mediated signaling, which unfold across three key research layers: elucidating IPMK’s cellular roles and mechanisms, assessing its physiological significance in vivo, and connecting these findings to disease processes. As HOIP and PIP3 are small molecules that bind to specific protein sites and modulate their activity, pinpointing their target proteins and characterizing these binding sites could pave the way for developing new synthetic drugs that block HOIP or PIP3 binding, thereby offering therapeutic benefits for disease treatment. Moreover, since HOIP is water-soluble, exploring its supplementation in food or developing in vivo delivery methods could be advantageous for treating diseases where IPMK deficiency is a primary factor.

## Data Availability

This is a review article, hence no original data are presented.
